# Limitations of human tau-expressing mouse models and novel approaches of mouse modeling for tauopathy

**DOI:** 10.3389/fnins.2023.1149761

**Published:** 2023-04-20

**Authors:** Naruhiko Sahara, Rin Yanai

**Affiliations:** Department of Functional Brain Imaging, Institute for Quantum Medical Sciences, National Institutes for Quantum Science and Technology, Chiba, Japan

**Keywords:** tau, tauopathy, FTDP-17 tau mutations, mouse model, neurofibrilary tangles

## Abstract

Neurofibrillary tangles (NFTs) composed of hyperphosphorylated tau protein are primarily neuropathological features of a number of neurodegenerative diseases, collectively termed tauopathy. There is no disease-modifying drug available for tauopathy except anti-amyloid antibody therapies for Alzheimer’s disease. For tau-targeting therapy, experimental models recapitulating human tau pathologies are indispensable. However, there are limited numbers of animal models that display intracellular filamentous tau aggregations. At present, several lines of P301L/S mutant tau-expressing transgenic mice successfully developed neurofibrillary pathology in the central nervous system, while most non-mutant tau-expressing transgenic mice rarely developed tau pathology. Importantly, recent studies have revealed that transgenes disrupt the coding sequence of endogenous genes, resulting in deletions and/or structural variations at the insertion site. Although any impact on the pathogenesis of tauopathy is unknown, gene disruptions may affect age-related neurodegeneration including tangle formation and brain atrophy. Moreover, some mouse lines show strain-dependent pathological features. These limitations (FTDP-17 mutations, insertion/deletion mutations, and genetic background) are a major hindrance to the establishment of a precise disease model of tauopathy. In this review, we noticed both the utility and the pitfalls of current P301L/S mutant tau-expressing transgenic mice, and we propose future strategies of mouse modeling to replicate human tauopathies.

## Introduction

The microtubule-associated protein tau is highly expressed in neurons and predominantly localized in axons (Binder et al., 1985). Mislocalized tau proteins abnormally aggregate into intracellular, filamentous inclusions (neurofibrillary tangles; NFTs) in brains of individuals with neurodegenerative diseases including Alzheimer’s disease (AD), progressive supranuclear palsy (PSP), corticobasal degeneration (CBD), argyrophilic grain disease (AGD), Pick’s disease, and familial frontotemporal lobar degeneration with underlying tau pathology (FTLD-Tau), all collectively referred to as tauopathies (Lee et al., 2001). To understand the regulatory mechanisms of tau-induced neurodegeneration, it is pivotal to establish precise disease models of tauopathy. Moreover, animal models of tauopathy are essential for the preclinical evaluation of drugs to prevent AD and related neurodegenerative diseases. The use of animal models that recapitulate the critical features of the diseases, such as NFTs, cognitive impairment, brain atrophy, and neuronal loss, is required. For successful modeling, multiple lines of P301L/S mutant tau-expressing transgenic mice (e.g., JNPL3, pR5, rTg4510, PS19, Line 2541) were generated, and they developed neurofibrillary pathology in the central nervous system in a promoter-dependent manner (Lewis et al., 2000; Gotz et al., 2001; Allen et al., 2002; Santacruz et al., 2005; Yoshiyama et al., 2007; Table 1). On the other hand, recent studies revealed that transgenes disrupt the coding sequence of endogenous genes, resulting in deletions and/or structural variations at the insertion site (Goodwin et al., 2019). Although any impact on the pathogenesis of tauopathy is unknown, gene disruptions may affect age-related neurodegeneration including tangle formation and brain atrophy. For example, integration sites of transgenes in the rTg4510 mice were recently identified (Gamache et al., 2019; Goodwin et al., 2019). In detail, human tau cDNA disrupted the first exons and promoter regions of fibroblast growth factor 14 (Fgf14) and CamK2a promoter-tetracycline activator transgene in Tg(CamK2a-tTA)1Mmay mice (CamK2a-tTA tg mice) (Mayford et al., 1996) disrupted *Vipr2, Wdr60, Esyt2, D430020J02Rik*, *Ncapg2* and *Ptprn2* genes. Notably, a knock-in mouse was generated with a single targeted *MAPT* cDNA transgene insertion in the downstream of collagen type I alpha I (Col1A1) to preclude the influence of Fgf14 deletion (Gamache et al., 2019). This mouse is called T2 mouse. The human tau transgene was expressed by breeding with the above mentioned CamK2a-tTA tg mouse. The authors clearly demonstrated that rTg4510 mice exhibited more pronounced forebrain atrophy than the newly developed rT2/T2 tauopathy mice. Since the rT2/T2 mice have an intact *Fgf14* gene, early pathogenesis of the rTg4510 mice may be caused by the loss of Fgf14 function. In addition, some mouse lines show strain background-dependent pathological features. Therefore, researchers must pay close attention to the handling of current mouse models of tauopathy in case there is a non-tau factor to induce neurodegeneration. In this review, we first discriminated the pathological phenotypes of tauopathy mouse models that are widely used for preclinical research projects. Next, perspectives of future mouse modeling will be proposed.

**Table 1:** List of tau transgenic mice.

Strain name	Isoform	Tau mutation	Promoter	Mouse strain	Pathological phenotype	Motor phenotype	Memory and cognitive performance	References	Insertion location	Refseq gene affected
Wild-type tau										
8c	Genomic human tau	Wild-type	Human tau	B6/D2/SW	Tau-ir axonal swellings			Duff et al. (2000)	ND	Trim30d
htau	Genomic human tau, mouse tau KO	Wild-type	Human tau	B6/129/SW	Neuronal loss, tau-ir pretangle and NFT in cortext & HP		Deficits on Morris water maze	Andorfer et al. (2003, 2005); Polydoro et al. (2009)	ND	Trim30d
ALZ17	2N4R	Wild-type	Mouse Thy1.2	B6/D2	Axonopathy, dystrophic neurites, gliosis in brain & SC	Motor defect		Probst et al. (2000)	ND	
htau40-1, 40–2, 40–5	2N4R	Wild-type	Mouse Thy1.2	FVB/N	tau-ir pretangles, axonopathy, dystrophic neurites, gliosis in brain &SC	Motor defect		Spittaels et al. (1999)	ND	
Wtau	2N4R	Wild-type	CaMKII	B6	tau-ir neuron and synapse loss in aged mice		Deficits on Morris water maze	Kimura et al. (2007)	ND	
rTg21221	0N4R	Wild-type	CaMKII-driven rTA + tetOp	FVB/129				Hoover et al. (2010)	chr2	
P301L/S tau										
JNPL3	0N4R	P301L	Mouse prion	B6/D2/SW	neuronal loss, gliosis & NFT in brain &SC, tau-ir glial inclusions	Motor defect		Lewis et al. (2000); Lin et al. (2003a,b)	ND	
pR5	2N4R	P301L	Mouse Thy1.2	B6/D2	tau-ir pretangles and NFT in brain & SC		Deficits on Morris water maze and conditioned taste aversion task	Gotz et al. (2001); Pennanen et al. (2004); David et al. (2005); Pennanen et al. (2006)	ND	
rTg4510	0N4R	P301L	CaMKII-driven rTA + tetOp	FVB/129	Neuronal loss, gliosis & NFT in limbic cortex		Deficits on Morris water maze	Santacruz et al. (2005); Spires et al. (2006); Sahara et al. (2013)	chr14	Fgf14
Tg tau(P301L) 23,027	2N4R	P301L	Hamster prion	FVB/129	Neuronal loss, gliosis & NFT in brain &SC, tau-ir glial inclusions		Deficits on Morris water maze, 8-arm radial maze, and conditioning taste aversion task	Murakami et al. (2006)		
tau-4R-P301L	2N4R	P301L	Mouse Thy1	FVB/N	tau-ir pretangles and NFT in brain & SC	Motor defect		Terwel et al. (2005)		
Line 2541	0N4R	P301S	Mouse Thy1.2	B6/CBA	Neuronal loss, gliosis & NFT in brain & SC	Motor defect		Allen et al. (2002)	ND	
PS19	1N4R	P301S	Mouse prion	B6/C3H	Neuronal loss, gliosis & NFT in brain	Motor defect		Yoshiyama et al. (2007)	chr3	None
VLW	2N4R	G272V, P301L, R406W	Mouse Thy1	B6/CBA	Dystrophic neurites, tau-ir pretangles, increased lysosomal bodies			Lim et al. (2001)	ND	
THY-Tau22	1N4R	G272V, P301S	Mouse Thy1.2	B6/CBA	Neuronal loss, gliosis & NFT in brain		Deficits on Morris water maze, increased anxiety on elevated plus maze	Schindowski et al. (2006)	ND	
Targeted mutation										
hTau-KI	Exon1-3 replaced to hTau	Wild-type	Mouse tau	C57BL/6 J				Saito et al. (2019)	chr11	MAPT
rT1	0N4R	Wild-type	CaMKII-driven rTA + tetOp	FVB/129			Deficits on Morris water maze	Gamache et al. (2020)	chr11	Col1A1
rT2 (rT2/T2)	0N4R	P301L	CaMKII-driven rTA + tetOp	FVB/129	Neuronal loss & NFT in limbic cortex		hyperactive in open field test	Gamache et al. (2019)	chr11	Col1A1

“tau-ir” means “anti-tau antibody immunoreactive.”

## Wild-type human tau-expressing mice without developing mature tangle formations

In adult human brain, six tau isoforms translated by the mRNA transcripts from alternative splicing of *MATP* gene are based on the number of microtubule-binding domains (3R and 4R tau) and the presence or absence of either one or two amino-terminal inserts (0 N, 1 N, or 2 N) (Goedert et al., 1989). AD tau aggregates composed of all six isoforms are dominated by paired helical filaments (Goedert et al., 1992), while straight filaments are present as the major morphology of tau filaments in FTLD-tau with 4R domains, such as PSP and CBD (Flament et al., 1991; Ksiezak-Reding et al., 1994), and Pick’s disease with 3R domains (Buee and Delacourte, 1999).

Human tau transgenic mice were generated by utilizing a cDNA construct to express wild-type tau under the control of a variety of promoters (Gotz et al., 1995; Brion et al., 1999; Ishihara et al., 1999; Spittaels et al., 1999; Probst et al., 2000; Higuchi et al., 2002). Wild-type 4R tau transgenic mice did not develop mature NFTs or robust neurodegeneration (Table 1). On the other hand, one of the wild-type 2N4R tau-expressing mice under control of the CaMKII promoter displayed age-dependent cognitive impairment and the corresponding neural activity change (Kimura et al., 2007).

The hTau model was generated by mating between the 8c mice that overexpress human tau derived from a P1-derived artificial chromosome (PAC) transgene containing the entire human *MAPT* gene (Duff et al., 2000) and tau knockout (KO) mice that have targeted disruption of exon one of *MAPT* (Tucker et al., 2001) to drive the expression of six human tau isoforms on a mouse tau KO background (Andorfer et al., 2003). The hTau mice express high levels of human 3R tau and relatively low levels of human 4R tau (Andorfer et al., 2003). This line shows an age-dependent tau pathology, DNA fragmentation, a quantitative loss of neurons, and spatial learning and memory deficits (Andorfer et al., 2003, 2005; Polydoro et al., 2009).

Recently, *MAPT* knock-in (KI) mice were generated by a homologous recombination approach that replaced the entire murine *MAPT* gene from exon 1 to exon 14 with the human ortholog (Saito et al., 2019). The *MAPT* KI mice expressed all six tau isoforms (Hashimoto et al., 2019), whereas adult wild-type mice only expressed 4R tau. In addition to the difference in isoform composition, the amino acid sequence in the N-terminal domain of murine tau is different from that of human tau while the C-terminal domain remains identical (Kampers et al., 1999). Although *MAPT* KI mice lacked apparent tau pathology and neurodegeneration, the authors revealed that tau humanization significantly accelerates pathological tau propagation of AD brain-derived tau aggregates. It should be noted that human tau expression was regulated by mouse promoter in the *MAPT*-KI mice while the PAC transgene in the hTau mice contains human tau promoter. Due to a lack of side-by-side comparison, differences of tau expression, isoform composition, and progression of tau pathology remain unclear.

rTg21221 mice expressing wild-type human 0N4R tau show equivalent levels of human tau protein to P301L human tau in rTg4510 mice (Hoover et al., 2010). Although an excessive level of human tau is conditionally expressed by regulation of CamK2a promoter, this line shows neither progressive memory deficits nor neurodegeneration. Of note, wild-type human tau mis-localization to dendritic spines was not observed, unlike P301L mutant tau.

Gamache et al. reported rT1 mice expressing nonmutant 0N4R human tau, which are genetically matched with rT2 mice with P301L tau mutation (Gamache et al., 2020). As mentioned previously, T1 and T2 mice were generated using site-specific targeting of transgene downstream of the Col1A1 3′ untranslated region. T1 and T2 mice were crossed to CamK2a-tTA tg mice, resulting in human 0N4R tau expression in forebrain neurons. The nonmutant 0N4R human tau protein level in rT1 mice was 12.6-fold of mouse endogenous tau. Human tau proteins extracted from 8-week-old rT1 mouse brains were highly phosphorylated in comparison with age-matched rT2 mice, while high molecular-weight tau species (64 kDa hyperphosphorylated tau) were not observed. Interestingly, the rT1 mice showed developmental-specific pathogenic properties, such as abnormal dynamics of human tau proteins, elevated tau protein stability and highly phosphorylated tau protein.

Overall, most wild-type human tau-expressing models are not feasible for therapeutic strategies because these models do not develop any neurofibrillary pathology, meaning that evaluation protocols are limited.

## P301L/S human tau-expressing mice developing mature tangle formations

Modeling of mature tau pathologies in mouse brains is crucial for therapeutic interventions. Until now, limited numbers of mouse models with mature tangles were successfully developed in a promoter-dependent manner. In FTDP-17 mutations, P301L and P301S mutations are often used in both *in vitro* and *in vivo* tau aggregation model systems probably because these mutations have the capability of filamentous formation, propagation properties, and low affinity to microtubules (reviewed in Gotz et al., 2018).

JNPL3 mice, which express P301L mutated human 0N4R tau under control of the mouse prion promoter, developed hind-limb dysfunction, and mature tangles concentrated in the brain stem and spinal cord (Lewis et al., 2000). About 50% neuronal loss in the spinal cord of JNPL3 mice was observed. Human tau protein levels and pathological tau accumulation progressed earlier in females than in males. Tau aggregates with 15–20 nm-wide straight or wavy filaments in neurons of JNPL3 mice were confirmed by immunoelectron microscopy (Lewis et al., 2000; Lin et al., 2003b). As a biochemical feature, the mice accumulated sarkosyl-insoluble, hyperphosphorylated tau that migrated to 64 kDa molecular size in SDS-PAGE (Sahara et al., 2002). The strain background of these mice significantly affects the phenotype. Male hemizygous JNPL3 mice on an in-bred C57BL/6 J strain at 15–18 months of age did not show any sign of motor deficits (Sahara et al., 2014a).

The pR5 mice that express P301L mutated human 2N4R tau under control of the mouse Thy-1.2 promoter were generated by another group (Gotz et al., 2001). These mice developed mature tau pathology from 8 months. Cognitive impairments were observed at 6 months and older (Pennanen et al., 2006).

Line 2541 mice, which express P301S mutated human 0N4R tau under control of the mouse Thy-1.2 promoter, were able to drive a similar pathology in both JNPL3 and pR5 mice (Allen et al., 2002). Biochemically, buffer-insoluble, hyperphosphorylated tau from the brain and spinal cord of line 2541 mice displayed identical mobility to the pathological tau from humans. Neurodegeneration in spinal cord motor neurons was extensive. Line 2541 mouse-derived dorsal root ganglion neurons showed phagocytosing live neurons with P301S tau aggregates and microglial hypophagocytic behavior, presumably inducing pathological tau propagation (Brelstaff et al., 2021).

**Figure 1:**
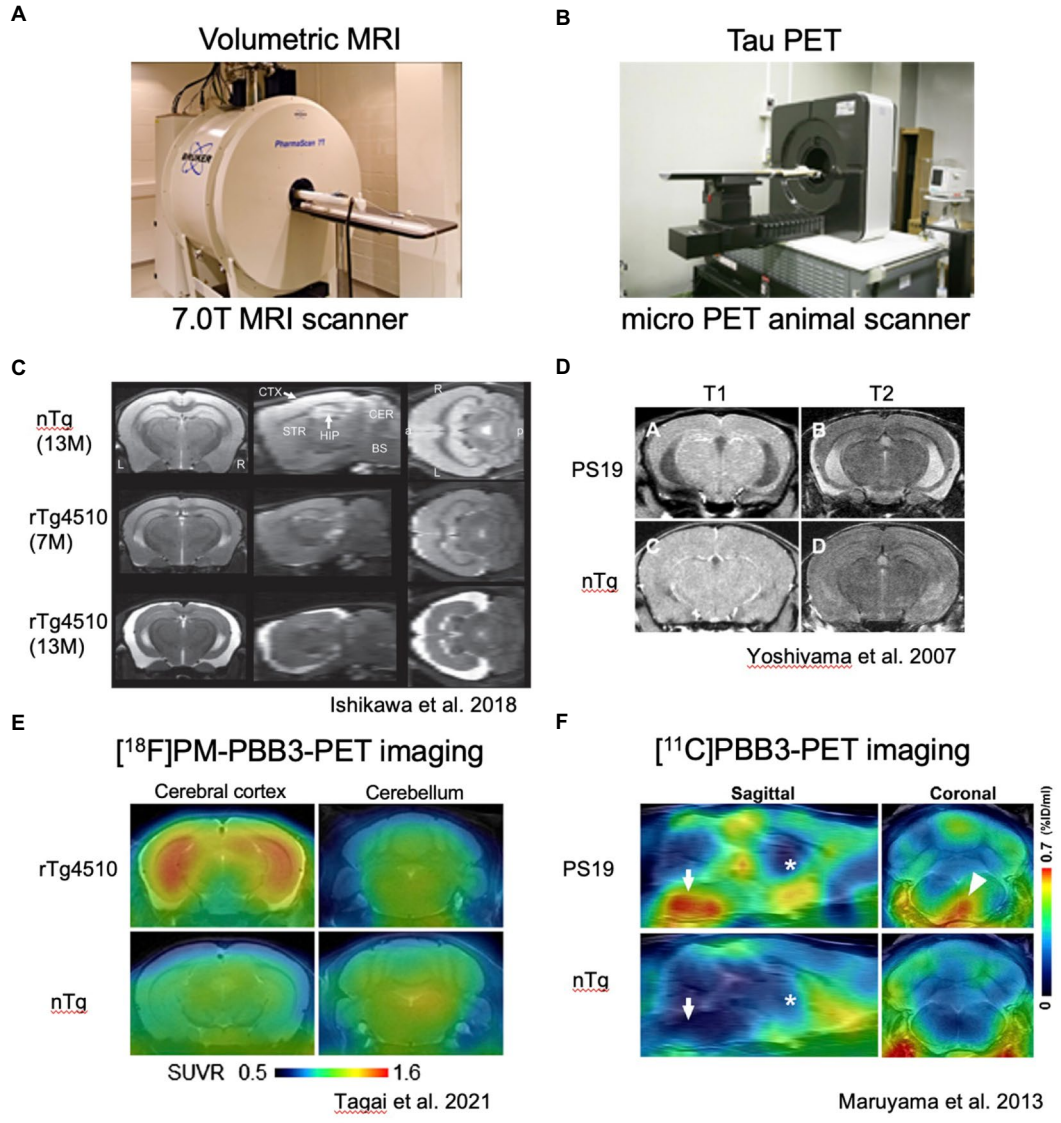
*In vivo* imaging analyses of rTg4510 and PS19 mouse brain pathologies. **(A)** Representative photograph of small animal MRI scanner. **(B)** Representative photograph of micro PET scanner. C and D. Volumetric MR images of rTg4510 mouse brains **(C)** with coronal, sagittal and horizontal slices and PS19 mouse brains **(D)** with coronal slices. E and F. Tau PET images of rTg4510 **(E)** and PS19 **(F)** mice. E. Coronal brain images of 9-month-old rTg4510 (top) and nTg (bottom) mice acquired by averaging dynamic PET data at 40–60 min after [^18^F]PM-PBB3 injections. **(F)** Sagittal and coronal PET images generated by averaging dynamic scans at 60–90 min after [^11^C]PBB3 injection.

The PS19 mouse line is another mutated human tau expressing model presenting mature tau pathology (Yoshiyama et al., 2007). These mice express P301S mutated human 1N4R tau under control of the mouse prion promoter. P301S tau expression caused pronounced neuronal loss in several brain areas as well as ventricular enlargement, as observed by volumetric magnetic resonance imaging (MRI) (Figures 1A,D). Impaired synaptic function, synapse loss and microglial activation preceded neurodegeneration by several months. Knockout of p62/SQSTM1, a ubiquitinated cargo receptor for selective autophagy, in PS19 mice significantly accelerates the progression of tau pathology (Ono et al., 2022), suggesting that p62-associated selective autophagy probably contributes clearance of pathological tau in this mouse model.

rTg4510 mice uniquely employed the CamK2a promoter-driven tetracycline effector transgene to express P301L mutated human 0N4R tau in the forebrain and provide conditional control of transgenic tau expression (Santacruz et al., 2005). The rTg4510 mice developed robust neurofibrillary pathology in the cortico-limbic area. The amount of P301L tau protein in the mouse forebrain at 2.5 months of age was about 13 times that of endogenous tau. rTg4510 mice develop pre-tangle pathology at 2.5 months and robust NFTs between 4 and 5.5 months (Ramsden et al., 2005). NFT is accompanied by decreasing solubility of P301L human tau, with a notable appearance of the 64 kDa tau band that co-migrates with hyperphosphorylated insoluble human tau. Approximately 50% of CA1 neurons are lost in 5.5-month-old rTg4510 mice (Spires et al., 2006). rTg4510 mice also showed profound deficits in cognition, thereby providing a robust behavioral measurement related to tau dysfunction, a quality that had not been shown in the rTg21221 mice expressing wild-type human tau (Hoover et al., 2010). Interestingly, suppression of P301L tau expression with doxycycline from ~13 times to ~3 times endogenous levels in rTg4510 mice reversed behavioral impairments in these mice although NFTs continued to increase, suggesting that NFTs were unlikely to be the initial neurotoxic species (Santacruz et al., 2005). Since the recent advances of *in vivo* imaging studies, tau pathology and brain atrophy can be captured in living rTg4510 mice (Sahara et al., 2017; Ishikawa et al., 2018) (Figure 1). Among the current tau positron emission tomography (PET) tracers, [^11^C]PBB3 and [^18^F]PM-PBB3 can detect non-AD tauopathy and rTg4510 tau pathology (Maruyama et al., 2013; Ishikawa et al., 2018; Tagai et al., 2021; Kimura et al., 2022; Figures 1B–F). In addition to tau PET imaging, manganese-enhanced MRI and diffusion tensor imaging (DTI) have been tested for the evaluation of brain functions and microstructural changes in living rTg4510 mice (Perez et al., 2013; Sahara et al., 2014b). Multimodal *in vivo* brain imaging rather than endpoint measurements must be an ultimate technique for investigating the real-time events of tauopathy.

## Similarity and difference in P301L/S human tau-expressing mice

**Figure 2:**
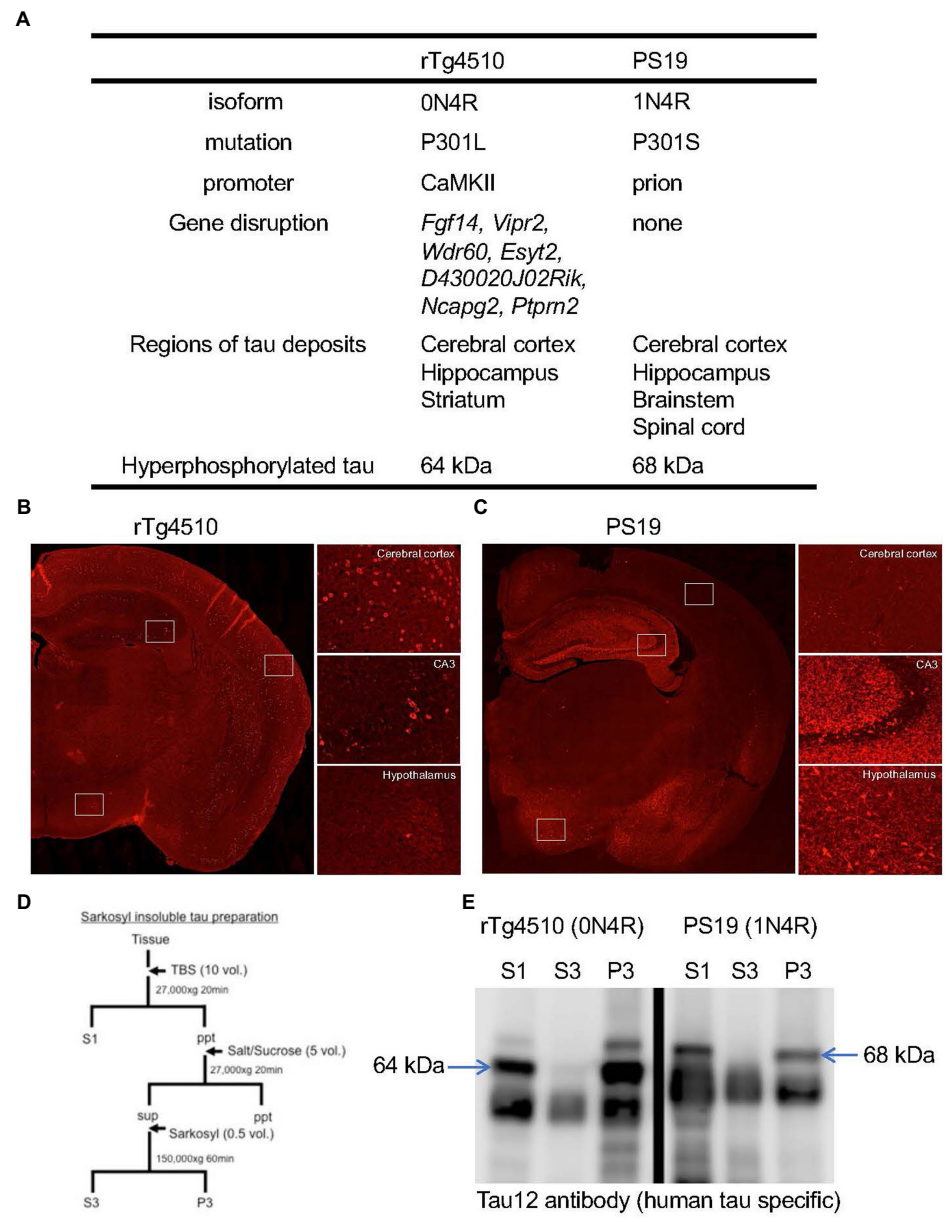
Pathological features of rTg4510 and PS19 mice. **(A)** Transgene constructs of tau gene, disrupted genes by transgene and distributions of tau pathology in rTg4510 and PS19 mice. **(B,C)** Tau immunohistochemistry on rTg4510 and PS19 mouse brain sections. Coronal sections of 7-month-old rTg4510 **(B)** and 12-month-old PS19 **(C)** mice were labeled with AT8 antibody. High-magnification views of cerebral cortex, hippocampus CA3, and hypothalamus were shown in the right panels. **(D)** Sample preparation protocol for the detection of tau protein from brain tissues. Tissues were separated into TBS-extractable (S1), high-salt and sarkosyl-soluble (S3) and sarkosyl-insoluble (P3) fractions. **(E)** Western blot of brain samples from 13-month-old rTg4510 and 10-month-old PS19 mice probed with human tau specific antibody, Tau12. Arrows indicate 64 kDa and 68 kDa hyperphosphorylated tau.

Human tau expression in both JNPL3 and PS19 mice is controlled by mouse prion promotor. The brain regions of human tau expression and tauopathy development were slightly different. Tau pathology in JNPL3 is more selective in the brainstem and spinal cord while that in PS19 appeared in the cerebral cortex, hippocampus, brainstem and spinal cord. Biochemically, both mice developed sarkosyl insoluble hyperphosphorylated tau (named 64 kDa and 68 kDa tau, respectively). Due to spinal cord pathology, both models developed hind-limb paralysis. Pathological progression in JNPL3 mice occurred under Swiss Webster background whereas that in PS19 developed under B6C3H or C57BL/6 J background. Progression of tau pathology in both models was varied among individuals within the same age group. In the JNPL3 mice, the severity of tauopathy was correlated with the human P301L protein level (Sahara et al., 2002). Although several factors (e.g., tau isoform, promotor, strain background) differ, the overexpression of P301L/S human tau most likely induces NFT formation and related brain atrophy (Table 1, Figure 2A).

PS19 mice exhibited accumulation of NFTs in the brainstem, but neuronal loss with few NFTs in the hippocampus (Ono et al., 2022) (Figures 1D, 2C). According to a previous report, postmortem brains of human FTDP-17 patients with P301S mutation showed weak tau immunoreactivity without NFTs due to the aggressive course of the disease (Lossos et al., 2003). Therefore, hippocampal pathology in PS19 is a better indicator for human P301S FTDP-17 pathology. However, it is unclear why regional differences of tau pathology between the brainstem and the hippocampus of PS19 mice exist. One possible explanation is that of a lower level of p62 in the hippocampus than in the brainstem for eliminating toxic and oligomeric species of tau protein (Ono et al., 2022). During the progression of neurodegenerative disease, selective neuronal and regional vulnerabilities are particularly important (Fu et al., 2018). Since pyramidal neurons in the entorhinal cortex and CA1 region of the hippocampus are more vulnerable during the process of AD pathology than caudal neurons in the spinal cord, pathological features in PS19 reasonably recapitulate the human tauopathy.

Similar to P301S mutation, P301L patients showed pretangle pathology in the frontal, temporal cortex, and dentate gyrus of the hippocampus (van Swieten et al., 1999). In contrast, both JNPL3 and rTg4510 mice expressing human P301L mutated 0N4R tau developed mature tangles confirmed by thioflavin S staining, Gallyas silver staining, and electron microscopy (Lewis et al., 2000; Santacruz et al., 2005). When the distributions of hyperphosphorylated tau inclusions between rTg4510 and PS19 mice were compared, clear differences were observed (Figures 2B,C). AT8-positive intraneuronal inclusions in rTg4510 mice revealed the entire cerebral cortex and pyramidal neurons of the hippocampus, while patchy and diffuse signals labeled by AT8 antibody were observed in the dentate gyrus, amygdala, and hypothalamus of PS19 (Figures 2B,C). In terms of biochemical properties, the hyperphosphorylated tau, which migrated to higher molecular size (64 and 68 kDa bands in Figures 2D,E) in SDS-PAGE, was recovered in sarkosyl-insoluble fractions from brain extracts of each mouse line (Figures 2D,E). This pool of tau was age-dependently increased in parallel with pathological tau accumulations (Sahara et al., 2013). Interestingly, tau pathologies in living PS19 and rTg4510 mice were successfully captured by the PET ligand PBB3 (Maruyama et al., 2013; Ishikawa et al., 2018; Ni et al., 2018) (Figures 1B–F). Since PBB3 can be used for fluorescence imaging, the spinal cord of a living PS19 mouse (12-month-old) and the somatosensory cortex of a living rTg4510 mouse (8-month-old) were directly scanned by two-photon microscopy after intravenous administration of PBB3 (Maruyama et al., 2013; Tagai et al., 2021). *In vivo* tau imaging is certainly a powerful tool for the therapeutic intervention of tauopathy.

## Neuroinflammation in P301L/S human tau expressing mice

It is well-known that both gliosis and neuroinflammation are prevalent in human tauopathies and mouse models (Leyns and Holtzman, 2017). As examples of tauopathy-related neuroinflammation models, PS19 mice under TREM2 knockout condition were previously examined (Leyns et al., 2017). Deficiency of TREM2 gene clearly reduced brain atrophy, tau-induced neurodegeneration and disease-associated microglial markers (Leyns et al., 2017). In PS19 mice, a deletion of complement component 3a receptor 1 (C3aR1) reversed neurodegenerative features, rescued tau pathology and reduced neuroinflammation (Litvinchuk et al., 2018). Astrocyte-released C3 also mediates the crosstalk between astrocytes and microglia, and C3 deficiency mitigates neurodegeneration and neuronal loss in PS19 mice (Wu et al., 2019). Deficiency of NOD-like receptor family pyrin domain-containing 3 (NLRP3) in PS19 mice significantly ameliorated tau-induced neurodegeneration and neuronal loss (Stancu et al., 2022). Overall, modulation of microglia activity works positively against tauopathy in PS19 mice.

Our recent study showed temporal change of microglial phenotype in both PS19 and rTg4510 mice using microglial markers. The mitochondrial 18-kDa translocator protein (TSPO), which is expressed in microglia, astrocytes and infiltrating immune cells in the central nervous system (CNS), is one of the inflammatory markers. Our group demonstrated longitudinal *in vivo* monitoring of tau pathology and TSPO accumulation in PS19 and rTg4510 mice using small-animal PET imaging (Maeda et al., 2011; Ishikawa et al., 2018). The age-dependent TSPO accumulation along with pathological tau accumulation and brain atrophy was confirmed by *in vivo* brain imaging studies. On the other hand, homeostatic microglia as a counterpart of the disease-associated microglial status were captured in these mouse models by anti-P2RY12 antibody. Immunohistochemical examinations in two distinct tauopathy mouse models revealed the reduction of P2RY12 in human tau expressing brain regions before pathological tau accumulation (Maeda et al., 2021). Microglial gene expression analysis in both PS19 and rTg4510 mice further confirmed the reduction of P2RY12 expression associated with tauopathy (Litvinchuk et al., 2018; Sobue et al., 2021).

Although It Is still under debate whether microglial activation Is a cause or a result of neurodegeneration, accumulating findings from tauopathy mouse models strongly support a linkage between neurodegeneration and neuroinflammation. Undoubtedly, The implication of microglia In neurodegenerative disorders Has recently attracted attention In terms of achieving effective therapies By The Use of neuroinflammatory targets.

## Next, generation models of tauopathy

During the development of transgenic mouse models, insertion of transgenes causes unexpected disruptions of the coding sequence of endogenous genes. In truth, insertions of human tau transgene and CamK2a-tTA transgene in the rTg4510 mice caused deletions of first exons and promoter regions of Fgf14 and disruptions of *Vipr2, Wdr60, Esyt2, D430020J02Rik*, *Ncapg2* and *Ptprn2* genes, respectively (Goodwin et al., 2019). In the case of PS19 mice, transgene was integrated into chromosome 3 and caused 249-kb deletion without affecting any reference gene (Goodwin et al., 2019). Since random integration may cause the unstable expression of transgenes and unpredictable phenotypes, it is preferable to insert target genes into the safe locus (Lombardo et al., 2011; Tasic et al., 2011). Based on this concept, a knock-in mouse was previously generated with a single targeted *MAPT* cDNA transgene insertion in the downstream of Col1A1 to preclude the influence of Fgf14 deletion (Gamache et al., 2019). After crossbreeding with CamK2a-tTA tg, resultant double transgenic rT2 mice expressed P301L mutated human 0N4R tau with 8.5 times of mouse endogenous tau, while the human tau protein level in rTg4510 mice was 12–13 times that of mouse endogenous tau. These protein levels cannot be explained by the number of transgenes (rT2 mice have one copy of *MAPT* cDNA transgene while rTg4510 mice have 70 copies of *MAPT* cDNA transgene). The rT2/T2 mice, which are homozygous of P301L mutated human 0N4R tau gene, express higher levels of protein than rTg4510 mice. However, progression of forebrain atrophy in the rT2/T2 mice was much slower than that in the rTg4510 mice. The difference in transgene integrations clearly confirmed the presence of intact Fgf14 gene in the rT2/T2 mice. The authors noticed that deletion mutation of Fgf14 may cause and/or accelerate tauopathy-like phenotypes in rTg4510 mice and cautioned against the use of rTg4510 mice for drug screening (Gamache et al., 2019). In the review by Di Re et al., Fgf14 belongs to the family of proteins interacting with voltage-gated Na + (Nav) channels at the axonal initial segment (Di Re et al., 2017). Fgf14 controls channel gating, axonal targeting and phosphorylation in neurons effecting excitability. F145S mutation in the Fgf14 gene was associated with spinocerebellar ataxia 27 (SCA27), one of the complex neurodegenerative disorders characterized by the onset of ataxia in early adulthood (van Swieten et al., 2003). Therefore, deficits of Fgf14 may cause neurodegenerative phenotypes, although further characterizations will be needed.

In CamK2a-tTA tg mice, it was reported that the tTA causes mouse strain-dependent neuronal loss in the dentate granule cell layer and behavioral alterations (Han et al., 2012). Doxycycline treatment causing tTA conformational change significantly prevents degeneration of the dentate granular cell layer, suggesting that tTA itself has a key potential role in inducing neurotoxicity. Since tTA-induced degeneration in the dentate granule cell layer was not evident in CamK2a-tTA tg mice under a congenic C57BL/6 J background, the C57BL/6 J strain may carry a recessive allele to protect against tTA-mediated physiological insult. Nevertheless, for generating conditional transgenic mice using a Tet-off system, both tTA overexpressing mice and tetracycline response element (TRE)-controlled transgene mice are preferable for maintaining under a C57BL/6 J strain background. In the future, a novel tTA expressing mouse without the transgene INDEL mutation will be needed to avoid disruptions of endogenous genes.

In our lab, we attempted a human tau transgene knock-in into the Rosa26 locus to generate a novel tauopathy mouse without any mutation in the *Fgf14* gene. The mouse Rosa26 locus showed ubiquitous transcriptional activity, but the loss of this gene is not lethal (Zambrowicz et al., 1997). This locus is widely used as a permissive site for targeted placement of transgenes in mice with no effect on animal viability or fertility (Soriano, 1999; Nyabi et al., 2009). Therefore, knock-in of a transgene into the Rosa26 locus enables locus-specific and copy number-controlled transgene expression. As a future plan, we will attempt to determine if an ideal mouse model generated by cross-breeding between Rosa26-Knock-in tau mice and CamK2a-tTA tg mice will induce tau overexpression and develop pathological criteria of tauopathy.

## Conclusion

Tauopathy is characterized by fibrillar tau accumulation in neurons and glial cells. In over a quarter of a century, we have made significant efforts for the development of tauopathy models in order to increase our understanding of the causal mechanisms of tauopathy-associated neurodegenerative disorders. Through careful investigations, a few mouse models expressing human tau with P301L/S mutation provided pathological criteria such as intracellular filamentous tau inclusions, biochemically confirmed hyperphosphorylated tau, neuronal loss, and brain atrophy. Since genetically engineered mouse models PS19 and rTg4510 are widely distributed throughout the world, these models significantly contribute to a general understanding of mechanistic studies and to the development of disease modifying therapies. However, traditional transgenic strategies cannot avoid unexpected disruptions of the coding sequence of endogenous genes. They happen to improve a tauopathy by modulating transgenic insertions without targeting tau-induced neurodegeneration. In this review, we propose a knock-in strategy into the safe locus and thereby maintain a certain strain background. Tetracycline-based inducible systems can be used to regulate temporal expressions of target genes (Navabpour et al., 2020). The rT2 mice have a single copy of human P301L tau transgene, but the level of human P301L tau protein was more than 8 times that of mouse endogenous tau protein. The control of a TRE in each insertion site might be better than the control of mouse tau promotor, although the exact mechanism still needs to be elucidated.

For future models, the generation of tau pathologies relevant to human tauopathies (e.g., AD, PSP, CBD, Pick, CTE and AGD) is feasible. Current advances with high-resolution cryoEM techniques clearly showed ultra-structures of tau fibrils at a near-atomic resolution (Shi et al., 2021). Tau filamentous ultra-structures of newly developed tauopathy mouse models can be evaluated more routinely in the future.

## Author contributions

All authors listed have made a substantial, direct, and intellectual contribution to the work and approved it for publication.

## Funding

This research was supported in part by grants from in-Aid for Science Research on Innovation Area (‘Singularity Biology’ 21H00446 to NS) and Scientific Research (C) (19K06896 to NS) from the Ministry of Education, Culture, Sports, Science, and Technology, Japan.

## Conflict of interest

The authors declare that the research was conducted in the absence of any commercial or financial relationships that could be construed as a potential conflict of interest.

## Publisher’s note

All claims expressed in this article are solely those of the authors and do not necessarily represent those of their affiliated organizations, or those of the publisher, the editors and the reviewers. Any product that may be evaluated in this article, or claim that may be made by its manufacturer, is not guaranteed or endorsed by the publisher.
